# Effect of Knowledge of Personal Metabolic-Trait SNP Genotypes with Tailored Lifestyle Recommendations on Body Weight and Body Composition: A Randomized Controlled Trial

**DOI:** 10.3390/nu18101550

**Published:** 2026-05-13

**Authors:** Jaewon Khil, Qiao-Yi Chen, Hyeonmin Gil, NaNa Keum

**Affiliations:** 1 Department of Food Science and Biotechnology, Dongguk University, Seoul 04620, Republic of Korea; kyk3079@naver.com (J.K.); chenqiaoyi0505@naver.com (Q.-Y.C.); 2 Department of Biomedical Informatics, Korea University College of Medicine, Seoul 02841, Republic of Korea; 3 Departments of Life Science, Pohang University of Science and Technology (POSTECH), Pohang 37673, Republic of Korea; gusals99@dgu.ac.kr

**Keywords:** knowledge of genetic risk information, tailored lifestyle recommendations, body composition, randomized controlled trial

## Abstract

Background/Objectives: Communicating genetic risk has been proposed as a motivational tool for weight control, but evidence remains limited. Methods: We conducted a randomized controlled trial among 53 overweight or obese young adults in South Korea. Participants were randomized to receive either their obesity-related genetic results with tailored lifestyle recommendations (intervention, *n* = 27) or genetic results limited to beauty traits (control, *n* = 26). Body weight and body composition were measured at baseline, 6 months, and 18 months. Primary outcome was change in body weight; secondary outcomes included body fat mass, body fat percentage, and skeletal muscle mass. Analyses used general linear and mixed models. Exploratory analyses examined effects among participants carrying ≥2 risk alleles across *FTO, MC4R*, and *BDNF*. Results: Overall, body weight and composition changes did not differ significantly between groups at 6 or 18 months. In exploratory subgroup analysis conducted among participants with obesity-related genetic risk, body weight increased in the intervention group (model-adjusted mean change, 2.68 kg; 95% CI, 2.28 to 3.09) but decreased in the control group (model-adjusted mean change, −11.58 kg; 95% CI, −1.99 to −1.18) over 18 months. Among participants with obesity-related genetic risk in the intervention group, those who reported behavior changes after receiving their genetic test results experienced modest weight reductions over 6 months compared with non-changers. Conclusions: Disclosure of obesity-related genetic information with tailored advice did not improve weight outcomes overall, but may benefit a subset of genetically susceptible individuals. Larger trials are warranted.

## 1. Introduction

The prevalence of obesity has reached epidemic proportions worldwide, with an estimated 58% of adults projected to be overweight or obese by 2030 [1]. In South Korea, the prevalence of obesity has also increased steadily, reaching approximately 38.4% in 2023 [2]. Because obesity is a well-established gateway to chronic diseases, including type 2 diabetes, cardiovascular disease, and certain cancers [3,4,5], there have been sustained efforts to identify effective lifestyle interventions for weight loss. Traditional randomized controlled trials (RCTs) have tested diverse strategies by modulating the amount of caloric intake [6], macronutrient composition [7], timing of intake [8], and the dose and delivery of physical activity [9], but long-term adherence and sustained weight loss remained elusive for many individuals [10]. These limitations highlight the need to move beyond one-size-fits-all approaches toward tailored, motivation-enhancing interventions.

With advances in genome-wide association studies (GWAS) [11], numerous common single-nucleotide polymorphisms (SNPs) implicated in obesity have been identified [12,13]. Among these, the rs9939609 variant in *FTO* is one of the earliest and most consistently replicated common genetic variants associated with body mass index (BMI) and obesity risk [14], and may influence food intake via hypothalamic appetite control [15]. Similarly, the rs17782313 variant near *MC4R*, a key gene in the melanocortin pathway involved in satiety signaling and energy homeostasis, has been associated with higher energy intake and obesity [16]. In addition, the rs6265 variant in *BDNF*, a functional polymorphism causing a valine-to-methionine substitution at codon 66 (Val66Met), has been linked to eating behaviors and BMI, potentially through the central regulation of appetite and energy balance [17,18,19,20]. Building on these findings, many nutrigenetic studies have examined how genetic variations modify individual responses to dietary interventions [21,22,23,24,25], thereby accumulating scientific basis for personalized diet prescriptions.

In parallel, direct-to-consumer (DTC) genetic testing has expanded rapidly, enabling individuals to access personal genetic information in a convenient and affordable manner. This development not only facilitates genotype-tailored counseling but may also create a “window of opportunity” in which receiving personal genetic-risk information motivates individuals to make appropriate behavioral changes, analogous to the way a cancer diagnosis can trigger risk-reducing lifestyle modifications [26,27]. Prior randomized controlled trials (RCTs) have reported modest changes in dietary intake or risk perception following genetic risk disclosure [28,29,30], but these studies have largely focused on short-term behavioral outcomes. In the Food4Me trial, which examined the effect of genetic-risk disclosure on weight-related outcomes, participants carrying the *FTO* risk allele who received genotype-based advice showed greater reductions in body weight and waist circumference at 6 months than those who were not informed of their genotype [31]. However, the trial relied on self-reported anthropometric outcomes, had a relatively short follow-up period, and restricted the intervention to *FTO* risk carriers. Therefore, we conducted an RCT to evaluate whether knowledge of personal obesity-related SNP results, accompanied by genotype-tailored lifestyle recommendations, influences objectively measured body weight and body composition over 18 months.

## 2. Method

### 2.1. Study Design

An RCT was conducted from June 2019 to November 2020 to examine the effect of knowing genetic disposition to obesity on weight management among overweight and obese individuals. The trial was conducted in accordance with the ethical standards of the Declaration of Helsinki and was approval by the Institutional Review Board of Dongguk University Ilsan Hospital (IRB number: 2018-12-006-010).

### 2.2. Study Participants

Between March and May 2019, participants were recruited through study information leaflets posted near Dongguk University Ilsan Hospital in South Korea. Eligible participants were men and women aged 18 years or older, who were overweight (BMI: 23–24.9 kg/m^2^) or obese (BMI: ≥25 kg/m^2^) by Asian BMI standards [32]. Exclusion criteria included a history of major disease or surgery, current use of prescription medications, mental illness, anemia, inability to exercise, and pregnancy or plans to become pregnant. A total of 53 individuals (32 men, 21 women) provided written informed consent to participate and were informed of their right to withdraw from the study at any time.

#### 2.2.1. SNP Testing

Prior to randomization, participants visited the research center where buccal swabs were collected to obtain oral epithelial cells. DTC genetic testing was performed through a commercial service (Theragen Bio, Seoul, Republic of Korea) [30], which provided information on selected genes related to metabolic health (e.g., obesity, blood glucose, blood cholesterol) and beauty traits (e.g., hair loss, skin elasticity). DNA was extracted using Exgene™ Tissue SV (GeneAll, Seoul, Republic of Korea), and SNP genotypes were identified with a SNP array (Theragen PMRA, Seoul, Republic of Korea). Details of SNP genotyping and quality-control procedures have been described elsewhere [33]. In Asian populations, the SNP array has demonstrated 94% accuracy for variants with minor allele frequencies greater than 5%.

The genetic testing results were accompanied by a user-friendly booklet containing corresponding lifestyle recommendations. For example, participants were genotyped for obesity-related variants including *FTO* rs9939609, *MC4R* rs17782313, and *BDNF* rs6265. For each SNP, carriers of the risk allele were encouraged to adopt a low-fat diet, to limit snacking, and to avoid emotional eating, respectively.

#### 2.2.2. Randomization

After the SNP testing results were obtained, participants were randomized in a 1:1 ratio with a computer-generated random number sequence. The intervention group received their complete SNP results, including obesity-related traits, along with corresponding lifestyle recommendations. In contrast, the control group was provided only with genetic test results related to beauty traits at baseline; information on metabolic health–related SNPs was disclosed only after the final follow-up assessment.

#### 2.2.3. Baseline and Follow-Up Measurements

Body weight and body composition (skeletal muscle mass, body fat mass) were measured using a bioelectrical impedance analysis device (InBody720, InBody Co., Ltd., Seoul, Republic of Korea) at baseline, 6 months, and 18 months, with participants wearing light clothing and barefoot. Height was assessed with an automatic stadiometer at baseline. Standardized self-administered questionnaires were completed by participants at baseline, 6 months, and 18 months to collect information on demographic characteristics, dietary intake (e.g., protein, carbohydrate, fat, alcohol, and other beverages), lifestyle factors (e.g., smoking status, physical activity), or changes in dietary and lifestyle habits in response to reading the genetic test report.

### 2.3. Statistical Analysis

The primary outcome was the change in body weight over 18 months, and secondary outcomes included changes in skeletal muscle mass and body fat mass over the same period. Analyses were conducted according to the intention-to-treat principle, with a complete-case approach for missing outcome data. To estimate the effect of knowing one’s obesity-related genetic risk on outcome changes at 6 and 18 months, general linear regression models were applied, adjusting for baseline outcome, baseline BMI, physical activity, smoking status and alcohol drinking. Participants with missing outcome data at each time point were excluded from the corresponding analysis. In addition, among participants who completed all follow-up visits, potential differences in the intervention effect over time were examined using linear mixed models that included an interaction term between group assignment and time. The aforementioned primary analyses were repeated separately in men and women, as responses to genetic information may differ by sex.

Of note, participants with genetic susceptibility to obesity may be more affected by learning their genetic information than those without such susceptibility. Thus, several exploratory analyses were conducted among participants with obesity-related genetic risk, defined as carrying ≥2 risk alleles across the three SNPs (*FTO* rs9939609, *MC4R* rs17782313, and *BDNF* rs6265). First, to examine whether knowing one’s genetic susceptibility to obesity has a more pronounced effect on weight management among participants with obesity-related genetic risk, we repeated the primary analyses in this subgroup. Second, to examine characteristics linked to behavioral responses to the genetic test report, we analyzed only participants with obesity-related genetic risk in the intervention group, who received personalized lifestyle recommendations. These participants were classified as “behavior changers” (those who reported modifying diet or lifestyle after reading the genetic test report at follow-up questionnaires) or “behavior maintainers” (those who reported no changes). Between the two groups, baseline characteristics were compared, and differences in outcome changes were evaluated using the same linear mixed model framework.

All *p* values were two-sided, and statistical significance was defined as *p* value < 0.05. Analyses were performed using SAS version 9.1 (SAS Institute, Inc., Cary, NC, USA).

## 3. Results

### 3.1. Characteristics of Study Population

**Figure 1:**
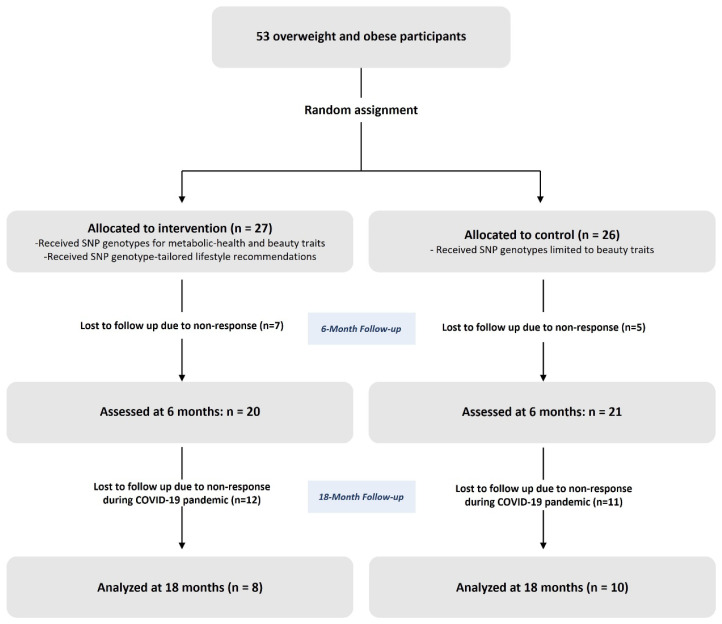
Flowchart of study participants.

A total of 53 overweight or obese young adults were randomized to the intervention (*n* = 27) or control group (*n* = 26) (Figure 1). Baseline characteristics of the participants had modest imbalances between the two groups (Table 1). The mean age was approximately 22 years, and about 60% were male. Compared with the control group, the intervention group had slightly lower body weight and body fat mass and percentage, whereas BMI and skeletal muscle mass were comparable. Approximately 58% of the participants reported ≥1 h/week of physical activity. The prevalence of current smoking was higher in the intervention group, whereas alcohol consumption was lower.

### 3.2. Effect of Knowledge of Personal Obesity-Related SNP Genotypes on Body Weight and Body Composition

**Figure 2:**
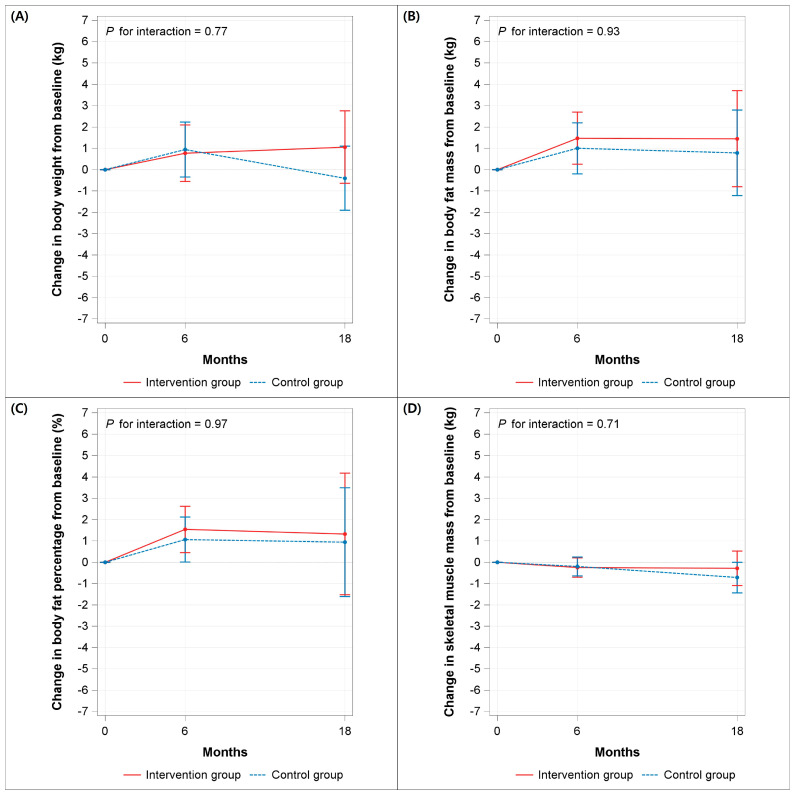
Changes in (**A**) body weight, (**B**) body fat mass, (**C**) body fat percentage, and (**D**) skeletal muscle mass from baseline to 6 and 18 months, by randomized group. Values are estimated marginal means and 95% confidence interval from general linear models, adjusted for baseline outcome, baseline body mass index, sex, physical activity, current smoking, and alcohol drinking. *p* values are for group x time interaction.

Changes in body weight did not differ significantly between the two groups at any time point (Figure 2A). From baseline to 6 months, the model-adjusted mean change was 0.77 kg (95% CI, −0.55 to 2.19) in the intervention group, and 0.94 kg (95% CI, −0.35 to 2.23) in the control group. From baseline to 18 months, the corresponding changes were 1.05 kg (95% CI, −0.64 to 2.75) and −0.40 kg (95% CI, −1.91 to 1.10), respectively. The effect of intervention on body weight did not vary by follow-up period (*p* for interaction = 0.77). A similar pattern was observed for changes in body fat mass (Figure 2B), body fat percentage (Figure 2C), and skeletal muscle mass (Figure 2D). Consistently, no significant between-group differences were observed at any time point for any outcome in either men or women (Supplementary Figures S1 and S2).

### 3.3. Effect of Knowledge of Personal Obesity-Related SNP Genotypes on Body Weight and Body Composition Among Individuals with Obesity-Related Genetic Risk

**Figure 3:**
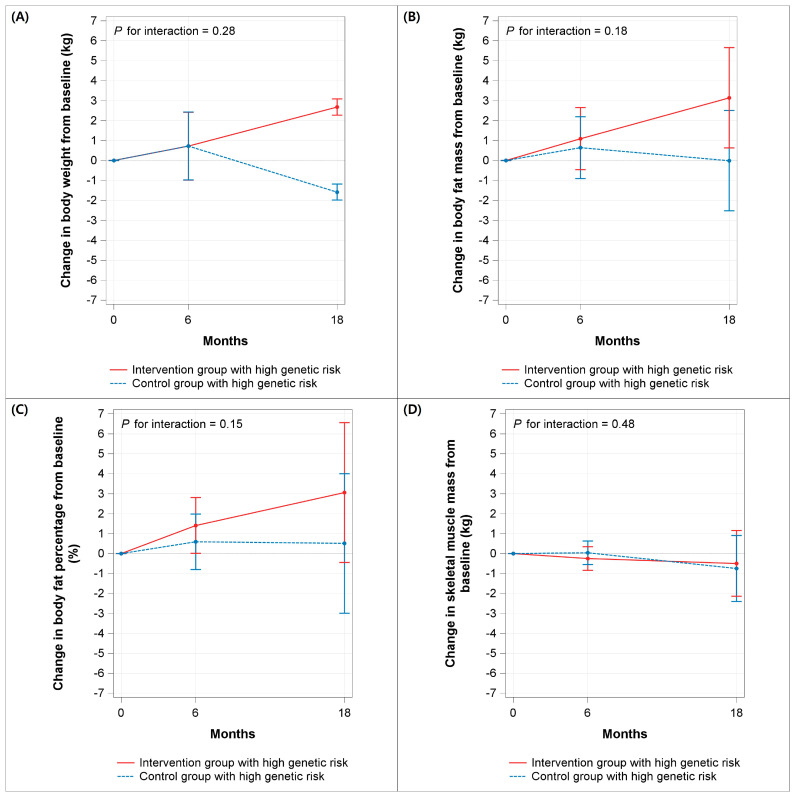
Changes in (**A**) body weight, (**B**) body fat mass, (**C**) body fat percentage, and (**D**) skeletal muscle mass from baseline to 6 and 18 months, by randomized group among participants with obesity-related genetic risk. Values are estimated marginal means and 95% confidence interval from general linear models, adjusted for baseline outcome, baseline body mass index, sex, physical activity, current smoking, and alcohol drinking. *p* values are for group x time interaction.

Among participants carrying ≥2 risk alleles across the three SNPs (*FTO* rs9939609, *MC4R* rs17782313, and *BDNF* rs6265), changes in body weight from baseline to 6 months did not differ significantly between the two groups, with the model-adjusted mean change 0.72 kg (95% CI, −0.98 to 2.42) in the intervention group and 0.73 kg (95% CI, −0.97 to 2.42) in the control group. From baseline to 18 months, however, the intervention group experienced the model-adjusted mean weight gain of 2.68 kg (95% CI, 2.28 to 3.09), which differed significantly (*p* < 0.001) from the model-adjusted mean weight loss of −1.58 kg (95% CI, −1.99 to −1.18) in the control group. The effect of intervention on changes in body weight did not vary by follow-up period (*p* for interaction = 0.28) (Figure 3A). There was no significant effect of intervention on changes in body fat mass (Figure 3B), body fat percentage (Figure 3C), and skeletal muscle mass (Figure 3D).

### 3.4. Characteristics of Behavior Changers and Maintainers Among Participants with Obesity-Related Genetic Risk in the Intervention Group

In the intervention group, 13 participants with obesity-related genetic risk completed the second questionnaire: 8 reported modifying diet or lifestyle after reading the genetic test, and 5 reported no changes (Table 2). Baseline characteristics differed between the two groups, with behavior changers having higher body weight, body fat mass, and body fat percentage than behavior maintainers. Behavior changers were also less likely to report ≥1 h/week of physical activity and more likely to be current smokers and drinkers. Compared with behavior maintainers, a greater proportion of behavior changers reported understanding the test results (100% vs. 40%) and perceiving the genetic test as helpful (88% vs. 40%). From baseline to 6 months, behavior changers showed a slight decrease in body weight (−0.09 kg), whereas behavior maintainers showed an increase (1.64 kg).

## 4. Discussion

In this RCT of 53 overweight or obese young adults, knowledge of personal obesity-related SNP information with tailored lifestyle recommendations was not associated with significant between-group differences in body weight or body composition changes at 6 and 18 months. Nevertheless, our exploratory subgroup analyses yielded interesting findings, although they should be considered preliminary and interpreted with caution due to the small sample size and substantial attrition. First, among participants with obesity-related genetic risk, the model-adjusted mean weight increased in the intervention group but decreased in the control group over 18 months, resulting in a statistically significant between-group difference. Second, among participants with obesity-related genetic risk in the intervention group, behavior changers had higher baseline adiposity and less healthy lifestyle profiles than behavior maintainers. They also more often reported understanding the genetic test report and perceiving it as helpful. The behavior changers showed slight weight reduction over time, whereas behavior maintainers did not. Overall, these exploratory findings should be considered hypothesis-generating rather than confirmatory.

Our primary finding of no long-term intervention effect aligns with results from prior RCTs evaluating genotype-based lifestyle interventions [34,35]. In the NOW trial conducted among overweight or obese adults in Canada, participants who received personal genetic information along with genotype-based lifestyle recommendations showed greater reductions in body fat percentage at 3 and 6 months, but no differences at 12 months in body fat percentage, weight, or BMI compared with those receiving standard lifestyle recommendations [34]. Similarly, in the MyGeneMyDiet^®^ trial conducted among overweight or obese Filipino adults over 12 months, no meaningful between-group differences were observed in anthropometric measures between participants informed of their genotype and genotype-tailored lifestyle recommendations and participants informed of their genotype but standard lifestyle recommendations [35]. Although these trials differ in intervention intensity and design, they consistently suggest that awareness of genetic susceptibility does not necessarily translate into sustained behavioral changes leading to improvements in adiposity [36]. Indeed, a meta-analysis of 18 RCTs reported no significant effects of DNA-based risk communication on diet, physical activity, or smoking cessation [37]. Nevertheless, in our exploratory subgroup analyses, genetically susceptible individuals with less healthy baseline lifestyles appeared more responsive to genetic risk disclosure, suggesting a potential role for targeted interventions in this subgroup.

Several factors may explain our overall null findings. From a psychological perspective, simply knowing one’s genetic predisposition may be insufficient to trigger meaningful behavior change [37]. While some participants may feel empowered to engage in weight control, others may react negatively or disengage, perceiving the risk as unmodifiable. Divergent responses—ranging from high-risk individuals acting proactively or adopting fatalistic attitudes, to low-risk individuals feeling reassured and making no changes or motivated by hope and a sense of agency—could offset each other, diluting the overall effect. In addition, a modest true effect could have been missed due to limited statistical power from the small sample size, and bias arising from selective attrition cannot be ruled out. Or possibly, the effect of knowledge of personal genotype and tailored lifestyles may be pronounced in a subgroup population. According to our findings, participants with obesity-related genetic risk and less healthy baseline lifestyles were more likely to modify their behaviors after receiving the genetic test report and showed a slight reduction in body weight over time.

Of note, among participants with obesity-related genetic risk, we observed an unexpected between-group difference: body weight increased in the intervention group but decreased in the control group. Given the small sample size, this finding is most likely attributable to chance variation (i.e., type I error). In addition, substantial loss to follow-up may have introduced bias if attrition was selective. Nevertheless, because psychological responses to genetic risk information, such as reduced perceived control, could theoretically discourage behavior change, a causal explanation cannot be entirely excluded. However, comprehensive reviews have reported that predisposing genetic test results have little effects on psychological outcomes, including perceived risk and perceived control over disease [36,38]. Thus, this subgroup finding should be considered exploratory and requires confirmation in future studies.

Our study has several limitations. First, the sample size was small (*n* = 53) and attrition was substantial—only 18 participants completed the 18-month follow-up—partly due to the COVID-19 pandemic during the follow-up period. These factors reduced statistical power to detect modest intervention effects and increased susceptibility to selection bias. In addition, although participants were analyzed according to their original randomized assignment, missing outcome data were handled using a complete-case approach. This may have reduced the benefits of randomization and introduced selection bias if participants who remained in the study differed systematically from those who were lost to follow-up. Therefore, our findings should be interpreted with caution. Second, due to the small sample size, baseline imbalances in lifestyle factors, including smoking and alcohol consumption, were observed despite randomization. Although we statistically adjusted for these factors, residual confounding cannot be ruled out, in part due to measurement error arising from self-reported assessment of lifestyle factors.

Third, the intervention centered on providing “knowledge” of personal genetic risk with brief genotype-tailored recommendations, but did not include ongoing behavioral support, feedback, or reinforcement. Prior research suggests that information alone is unlikely to produce durable behavior change without structured guidance or reinforcement [39,40]. Therefore, the null overall findings in our study might reflect the limited intensity of the intervention rather than the absence of any potential role for genotype-tailored risk communication. Finally, participants were relatively young and self-selected, which may limit generalizability to older adults, individuals at higher disease risk (e.g., those with a strong family history), or highly health-conscious populations who may engage more actively with genetic information.

Yet, our study has several strengths. To the best of our knowledge, this is the first RCT to evaluate the effects of awareness of obesity-related genetic risk in a real-world DTC setting, integrating genotype-tailored lifestyle recommendations along with self-reported behavioral responses and objectively measured body composition. With longitudinal data up to 18 months, our study captured both short-term and long-term effects of the intervention. Exploratory subgroup analyses, which were restricted to individuals with genetic vulnerability to obesity and compared behavior changers with maintainers in response to the personalized intervention, offer insights into individual variability in response to communicating personal genetic information. These observations may inform the design of future genotype-tailored, motivation-enhancing interventions and guide DTC genetic testing services seeking to translate genetic information into meaningful lifestyle change and improved health. 

## 5. Conclusions

In conclusion, knowledge of personal obesity-related SNP information accompanied by genotype-tailored lifestyle advice did not yield significant improvements in body weight and body composition among overweight or obese individuals. Exploratory findings suggest potential for effectiveness in subgroups, with individuals who have higher genetic risk and less healthy lifestyles appearing more responsive to genetic risk information. In the context of growing interest in personalized nutrition and the increasing burden of obesity, these findings suggest that genetic risk disclosure alone may be insufficient as a population-level strategy but may have value when targeted to individuals most likely to engage with such information. Larger, well-conducted trials with structured behavioral support are needed to identify responsive subgroups and clarify the role of genotype-tailored interventions in obesity management.

## Figures and Tables

**Table 1:** Baseline characteristics of the study participants by randomized assignment.

	Intervention Group (*n* = 27)	Control Group (*n* = 26)
**Body weight, kg**	74.3 (12)	76.8 (11)
**BMI, mean, kg**	25.9 (4)	26.1 (3)
**Body fat mass, kg**	21.1 (10)	23.1 (7)
**Body fat percentage, %**	28.3 (11)	31.1 (8)
**Skeletal muscle mass, kg**	29.8 (7)	30.1 (6)
**Age, years**	22.2	22.3
**Men, *n***	16 (59)	16 (61)
**Physical activity, *n***		
**<1 h/week**	11 (41)	11 (42)
**≥1 h/week**	16 (59)	15 (58)
**Currentsmoking, *n***		
**No**	20 (74)	23 (88)
**Yes**	7 (26)	3 (12)
**Alcoholdrinking, *n***		
**Almost never**	14 (52)	11 (42)
**≥1 time/week**	13 (48)	15 (58)

Data are *n* (%) or mean (SD) unless otherwise indicated.

**Table 2:** Characteristics of participants with obesity-related genetic risk in the intervention group, by the status of behavioral change.

	Behavior Changers (*n* = 8)	Behavior Maintainers (*n* = 5)
**At baseline**		
**Body weight, kg**	74.5 (6)	71.8 (10)
**BMI, kg**	25.1 (1)	24.5 (1)
**Body fat mass, kg**	19.1 (7)	16.1 (5)
**Body fat percentage, %**	25.8 (9)	23.5 (10)
**Skeletal muscle mass, kg**	31.0 (6)	31.4 (9)
**Age, years**	22.0	21.4
**Men, *n***	5 (62)	3 (60)
**Physical activity, *n***		
**<1 h/week**	2 (25)	1 (20)
**≥1 h/week**	6 (75)	4 (80)
**Currentsmoking, *n***		
**No**	5 (62)	4 (80)
**Yes**	3 (38)	1 (20)
**Alcoholdrinking, *n***		
**Almost never**	2 (25)	3 (60)
**≥1 time/week**	6 (75)	2 (40)
		
**At 6-month**		
**Perceived understanding of genetic testing report, *n***		
**Low**	0 (0)	3 (60)
**High**	8 (100)	2 (40)
**Perceived helpfulness of genetic testing, *n***		
**No**	1 (12)	3 (60)
**Yes**	7 (88)	2 (40)
**Change in body weight from baseline, kg**	−0.09 (2)	1.64 (2)

Data are *n* (%) or mean (SD) unless otherwise indicated.

## Data Availability

The data are not publicly available due to privacy and ethical restrictions. Requests for access to these data can be directed to the corresponding author and will be considered upon reasonable request.

## Supplementary Materials

The following supporting information can be downloaded at: https://www.mdpi.com/article/10.3390/nu18101550/s1, Figure S1: Changes in (A) body weight, (B) body fat mass, (C) body fat percentage, and (D) skeletal muscle mass from baseline to 6 and 18 months, by randomized group in men; Figure S2: Changes in (A) body weight, (B) body fat mass, (C) body fat percentage, and (D) skeletal muscle mass from baseline to 6 and 18 months, by randomized group in women; Table S1: Baseline characteristics of study participants by randomization group and sex.
