# Supplementary material for: Effect of Knowledge of Personal Metabolic-Trait SNP Genotypes with Tailored Lifestyle Recommendations on Body Weight and Body Composition: A Randomized Controlled Trial
Source: Nutrients. 2026 May 13;18(10):1550. doi: 10.3390/nu18101550 (PMC13209989; doi:10.3390/nu18101550)
Supplement: Supplementary file 1 [file nutrients-18-01550-s001.zip › nutrients-4259358-supplementary.pdf]

Table S1. Baseline characteristics of study participants by randomization group and sex.

	Men (n= 32)		Women (n= 21)	
	Intervention group	Control Group	Intervention group	Control Group
**N**	16 (50)	16 (50)	11 (52)	10 (48)
**Body weight, kg**	79.5 (12)	82.7 (10)	66.8 (8)	67.4 (5)
**BMI, mean, kg**	25.9 (4)	26.4 (4)	25.8 (4)	25.6 (2)
**Body fat mass, kg**	18.0 (10)	22.0 (8)	25.5 (7)	24.9 (4)
**Body fat percentage, %**	21.8 (8)	26.0 (6.6)	37.7 (6)	36.7 (4)
**Skeletal muscle mass, kg**	34.9 (3)	34.3 (3)	22.4 (2)	23.3 (1)
**Age, years**	22.9	23.3	21.2	20.7
**Physical activity, n**				
**<1hour/week**	7 (44)	6 (38)	4 (36)	5 (50)
**≥1hour/week**	9 (56)	10 (62)	7 (64)	5 (50)
**Current smoking, n**				
**No**	9 (56)	14 (88)	11 (100)	9 (90)
**Yes**	7 (44)	2 (12)	0 (0)	1 (10)
**Alcohol drinking, n**				
**Almost never**	4 (25)	6 (38)	10 (91)	5 (50)
**≥1time/week**	12 (75)	10 (62)	1 (9)	5 (50)

Data are n (%) or mean (SD) unless otherwise indicated.

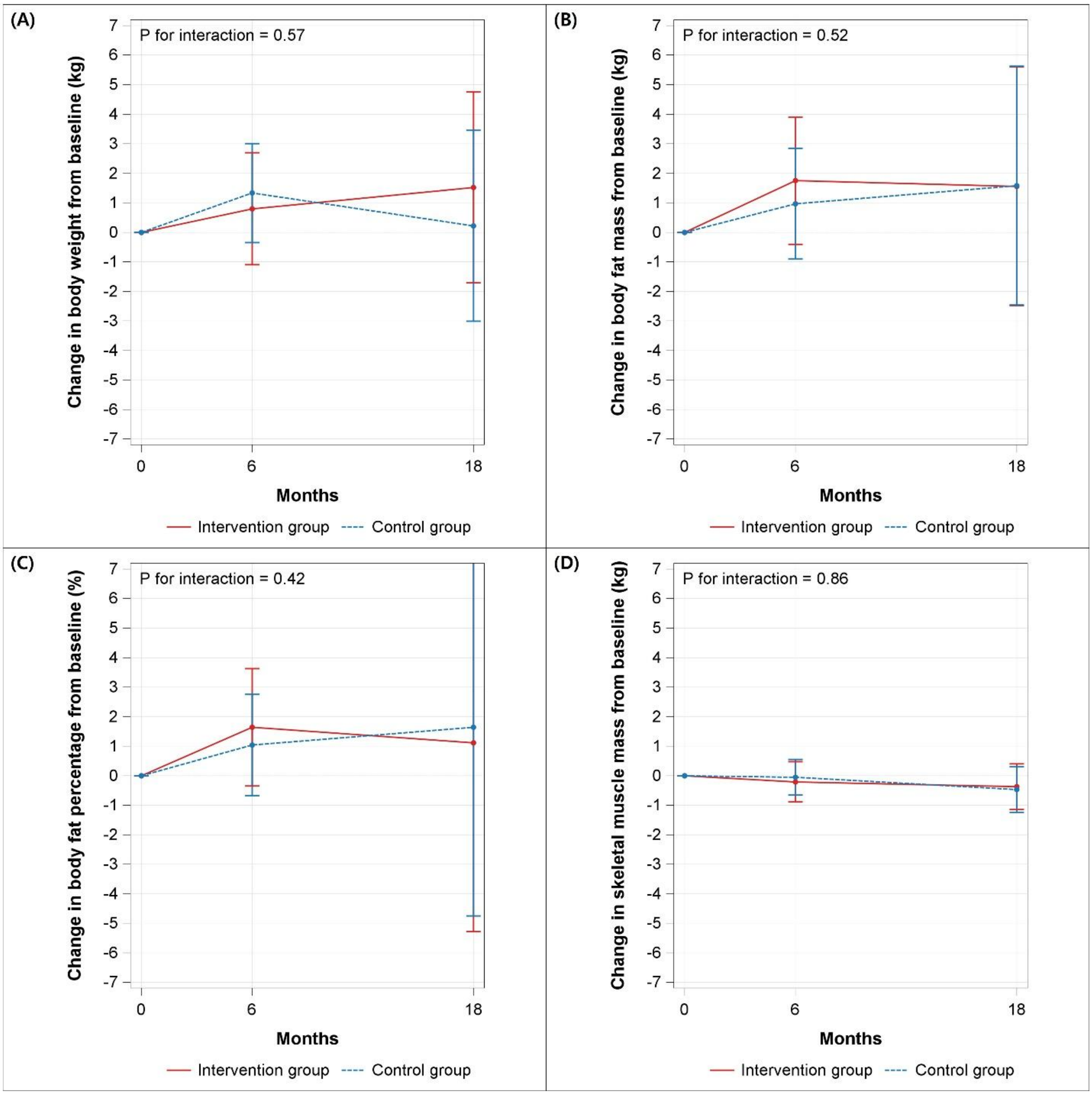

Figure S1. Changes in (A) body weight, (B) body fat mass, (C) body fat percentage, and (D) skeletal muscle mass from baseline to 6 and 18 months, by randomized group in men. Values are estimated marginal means and 95% confidence interval from general linear models, adjusted for baseline outcome, baseline body mass index, physical activity, current smoking, and alcohol drinking. P values are for group x time interaction.

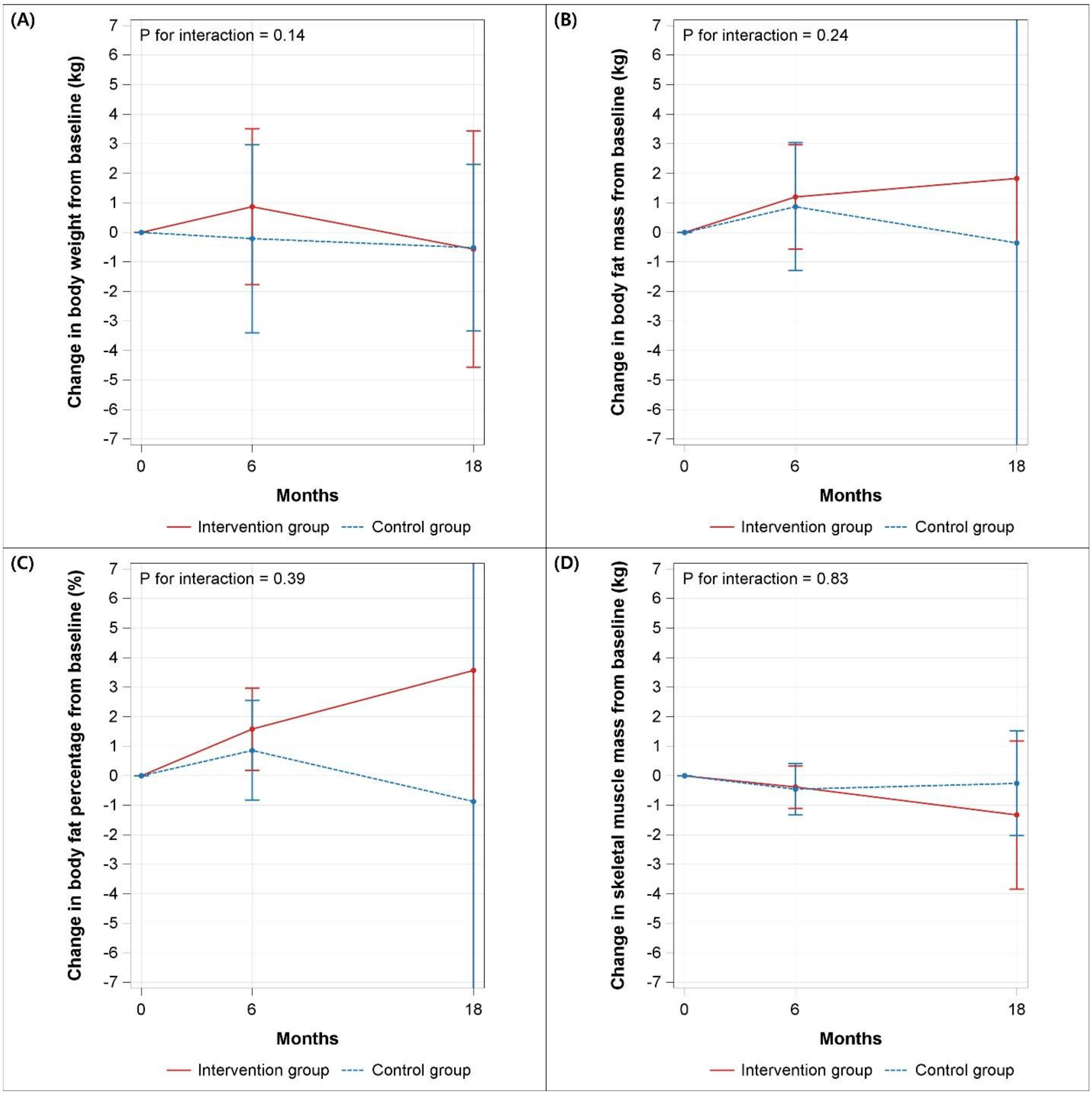

Figure S2. Changes in (A) body weight, (B) body fat mass, (C) body fat percentage, and (D) skeletal muscle mass from baseline to 6 and 18 months, by randomized group in women. Values are estimated marginal means and 95% confidence interval from general linear models, adjusted for baseline outcome, baseline body mass index, physical activity, current smoking, and alcohol drinking. P values are for group x time interaction.
